# Use of *Trichoderma* in the Production of Forest Seedlings

**DOI:** 10.3390/microorganisms12020237

**Published:** 2024-01-23

**Authors:** Natália Cássia de Faria Ferreira, Maria Lucrecia Gerosa Ramos, Alcides Gatto

**Affiliations:** 1 Department of Forestry Engineering, Faculty of Technology, University of Brasilia, Brasilia 70910-900, DF, Brazil; natcassiadefaria@gmail.com; 2 Faculty of Agronomy and Veterinary Medicine, University of Brasilia, Brasília 70910-900, DF, Brazil; lucreciaunb@gmail.com

**Keywords:** plant–microorganism interaction, synergism, growth promotion

## Abstract

Forest production has great relevance in the Brazilian economy, characterized by several production sectors, including the production of seedlings. With the focus on maximizing the capacity of survival, development, and adaptation of seedlings, *Trichoderma* is highlighted as a potentially useful genus of microorganisms for promoting growth and higher product quality. In this sense, this review aims to describe the main mechanisms of fungi action in forest seedlings’ production. The different species of the genus *Trichoderma* have specific mechanisms of action, and the current scenario points to more advances in the number of species. The interaction process mediated by different mechanisms of action begins in the communication with plants, from the colonization process. After the interaction, chemical dialogues allow the plant to develop better because, from colonization, the forest seedlings can maximize height and increase shoot and root development. Fungi promote solubilization and availability of nutrients to seedlings, which show numerous benefits to the development. The use of beneficial microorganisms, such as fungi of the genus *Trichoderma*, has become a sustainable strategy to enhance seedling development, reducing the use of agrochemicals and industrial fertilizers.

## 1. Introduction

Forest production has great relevance in the Brazilian GDP, characterized by several production sectors, including seedlings. Despite the global scenario of the COVID-19 pandemic (SARS-CoV-2), the forest production chain showed resilience in the Brazilian market, with a growth of 7.5% in 2021, higher than the evolution of the national GDP [1]. This sector has great economic importance, intended for commercial plantations and recovery plans of degraded areas, which denotes the need to expand the capacity of seedling production and obtain higher production rates [2].

Despite the relevance of the forestry sector, in Brazil, there is still a great dependence on the import of inputs, with emphasis on synthetic fertilizers, which requires measures correlated with the reduction in the use of fertilizers and adequate practices to obtain success in the production of seedlings [3]. The production of woody plants promotes environmental and economic benefits by reducing the use of chemicals, including having less soil and water contamination, having sustainable production, spending less time on the formation of seedlings, and having higher-quality parameters [4].

To obtain high-quality seedlings, it is necessary to achieve high growth parameters arising from morphological and physiological attributes that reflect the development capacity and survival in the field. The morphological attributes are used to evaluate the ability of plant development, measured by height, the diameter of the neck, and root development. In contrast, the physiological attributes are obtained through the capacity of absorption of water and nutrients by the plant to provide important information on the performance of the species and efficiency in the production of seedlings, because vigorous seedlings with high field performance are defined in the initial phase of seedling establishment and development [5,6].

However, there are gaps in adopting technologies that increase biomass production with lower costs and production time. There are technologies aimed to improve the germination potential of seeds. To maximize the survival, development, and adaptation capacity of seedlings, the inoculation of microorganisms can improve growth and higher production quality [7,8].

When inoculated in seedlings and after the colonization of the root system by *Trichoderma*, changes occur in plant metabolism and increase root development, growth, and nutrition of plants [9]. The main mechanism of interaction between *Trichoderma* and seedlings occurs via chemical signaling, resulting from the production of compounds responsible for modifications in the transcriptome, proteome, and plant metabolome [10].

From there, the beneficial action of fungi begins via the modulation of molecular centers and prolonged systemic responses that stimulate plant development [11,12]. As there are broad benefits promoted by fungi of the genus *Trichoderma* in plant development, the objective of this review was to discuss the main mechanisms of action of fungi in the production of forest seedlings.

## 2. Fungi of the Genus *Trichoderma*: Beneficial Microorganisms

The genus *Trichoderma* is the imperfect phase of Hypocrea, belonging to the Kingdom Fungi, Phylum Ascomycota, Class Sordariomycetes, Order Hypocreales, and Family Hypocreaceae [13]. It comprises many species of free-living filamentous fungi in multiple ecosystems, from tropical to temperate regions, characterized by accelerated growth. They are considered highly active species in the soil, associated with the rhizosphere and the decomposition process of plant residues and wood, and are rarely associated with plant diseases [14].

The fungi *Trichoderma* spp. have bright green conidia and a repeatedly branched conidiophore, are opportunistic and avirulent plant symbionts, and have asexual reproduction by the production of conidia and chlamydospores and in wild habitats by ascospores [9]. In recent years, there has been an exponential increase in the number of species identified in the genus, and the current scenario points to further advances in the number of species [15].

## 3. Interaction Process: *Trichoderma* spp. and Forest Species

During the production process, the seedlings are generally subjected to several abiotic factors (climate, temperature, water availability) and/or biotic stress (phytopathogenic agents). These stresses may lead to considerable restrictions to obtain vigorous seedlings with high-quality indexes. The inoculation of *Trichoderma* fungi enhances acclimatization, resistance/tolerance through plant response mechanisms under adverse conditions and, above all, induces greater growth of seedlings [16,17,18].

The colonization of roots results in physical or biochemical responses, initiating chemical transmission, in which plants produce secondary metabolites (SM), constituting limiting factors for the invasion of the fungus in the cortical cell layers in the roots [19]. According to the barriers imposed by plants, *Trichoderma* fungi can produce strategies to “dribble” plant responses; this occurs via hormone production to establish a prolonged mutualistic association without the occurrence of obstacles that can make the root establishment and the symbiotic process unfeasible [10].

**Figure 1:**
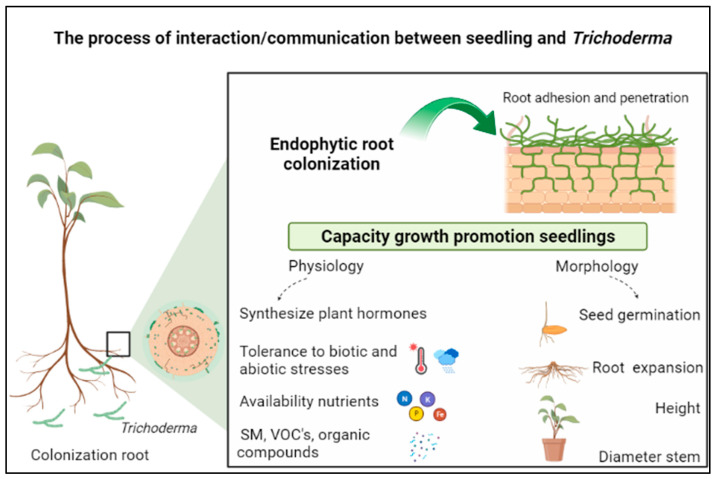
Interaction processes between *Trichoderma* spp. and forest seedlings. Created in Biorender.

The interaction process between *Trichoderma* and plants is mediated by different mechanisms of action that begin in the communication with plants from the root colonization process. Figure 1 illustrates the stages of the interaction process between *Trichoderma* spp. and the host plant, starting via the release of root exudates, recognized by the fungus and responsible for chemotaxis (release of secondary metabolites). Subsequently, the fungus adheres to the root surface via protein action (hydrophobins) and produces enzymes responsible for cell degradation (cellulolytic, proteolytic, pectinolytic, and xylanolytic) and colonization of the root epidermis and cortex.

## 4. Potential Mechanisms of Interaction

*Trichoderma* fungi cause physiological changes and plant metabolism when colonizing the root system. From a practical point of view, the interaction is promising since it allows high-quality indexes of seedlings to be achieved, according to the release of compounds, which the root system will assimilate, solubilizing and absorbing nutrients by plants [9].

The ability to synthesize plant hormones is commonly observed in *Trichoderma* species [20,21]. Auxins, abscisic acid, cytokinin, ethylene, and gibberellins stimulate plant growth, especially in adverse conditions [22]. The biosynthesis of indole-3-acetic acid (IAA) performed by the fungus increases root development and production of secondary roots and root hairs [23].

The expansion of the root system occurs through endophytic colonization and the production of phytohormones in the induction of water and nutrient efficiency by plants, in addition to greater tolerance to biotic and abiotic stresses [24]. In the dynamic plant–microorganism communication process, the interaction with roots and other parts of the plant can be influenced by different factors, such as soil type, the potential of strains, and plant species [25].

### 4.1. Solubilization and Availability of Mineral Nutrients

Despite the high nutritional requirement of forest seedlings, Brazilian soils generally have low natural fertility and a high degree of weathering, which limit obtaining greater production potential since the low availability of nutrients in the soil reduces the efficiency of absorption and use of macro and micronutrients by plants [26]. Considering that the soil composition is a fundamental part of the forest production system, it is important to adopt management practices to maintain its physical–chemical–biological quality and obtain vigorous, productive, and profitable woody plants [27].

In general, the mineral nutrients available to plants cannot meet the appropriate demands in the production of vigorous seedlings; this factor is associated with the existence of and functions performed by microorganisms in the soil [28]. In this sense, a strategy to reduce the nutritional restriction to the production of seedlings becomes necessary to potentiate the production. Therefore, the adequate absorption of nutrients is important during the initial phase of seedling formation, with emphasis on phosphorus (P) because the insufficient nutrition restricts the production and quality of plants [29].

The macronutrient phosphorus (P) in soils occurs in two forms: organic (plant decomposition) and inorganic (salts such as calcium (Ca), iron (Fe), and aluminum (Al)). However, most of it is in the inorganic and insoluble form, making its availability to plants unfeasible [30]. Phosphate rock is the world’s main P source; however, seedlings’ production depends on the continuous supply of phosphate fertilizers [31]. In this scenario, aiming at less dependence, there is a growing need to seek and adopt sustainable strategies capable of improving P availability [32].

Fungi release substances such as volatile organic compounds (VOCs) and SM to form complexes with Fe (III) or reduce such element [33]. Fe is solubilized by siderophores and changes root morphology via induction of root hairs, which enables the absorption of this micronutrient, and this process is involved in several metabolic processes [34,35]. These mechanisms are the most common among *Trichoderma* fungi; however, the responses can be variable according to the capacity of each strain [36].

**Figure 2:**
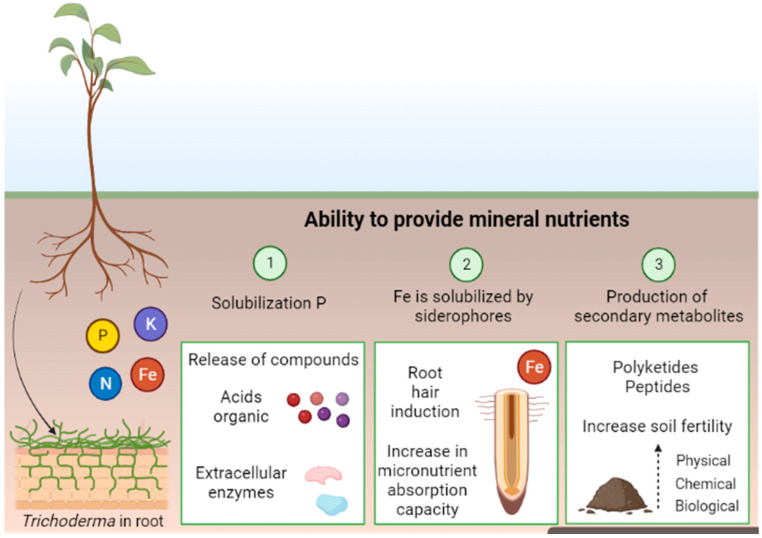
The mechanisms by fungi of the genus *Trichoderma* in association with woody plant species. Created in Biorender.

The nutritional flow system is mediated by complex interactions influenced by chemical reactions between the root system and *Trichoderma* fungi. The strains of the genus *Trichoderma* have different mechanisms of action on soil nutrients; however, the ability to solubilize and mineralize P is emphasized, transforming it into soluble forms for plant absorption [37,38]. Figure 2 illustrates the interaction process that is coordinated by multiple actions of microorganisms in the solubilization of P from biochemical mineralization (enzymatic release) and secretion of chemical complexes responsible for mineral solubilization (siderophores, protons, hydroxyl ions, organic acids), increasing P absorption by seedlings [39,40,41,42].

In addition to organic compounds, the nutritional availability reaction can occur by producing secondary metabolites (SM), such as polyketides and peptides, which increase soil fertility [42]. It is worth mentioning that the greater the ability to obtain resources from the root system, the greater the development and survival of seedlings in the field [43]. Plants more efficient at absorbing water and nutrients can increase photosynthetic potential, achieving higher root systems [44].

### 4.2. Production of Organic Compounds, Secondary Metabolites, and Plant Hormones

The chemical signals released from the interaction between plant and *Trichoderma* produce complex association responses [45]. However, the effects obtained depend on the ability of *Trichoderma* strains to act on the production of chemical compounds, which promote biochemical changes in plants (Table 1).

Furthermore, the production of chemical compounds is influenced by environmental conditions, and temperature, humidity, and soil pH are determining factors, considering that each species of *Trichoderma* produces different types of compounds and positively affects plant growth [49]. The VOCs belong to several chemical classes: mono and sesquiterpenes, alcohols, ketones, lactones, esters, phenols, thioesters, and cyclohexenes [56]. Fungi can produce different types of VOCs used to promote plant growth [57]. This process occurs from producing bioactive compounds and modulation of plant hormones, with benefits to increase root volume, plant biomass, and productivity [58,59].

## 5. Promoting the Growth of Forest Species

The development of seedlings depends on the adequate management of essential resources to plant functioning, and the phase of establishing of the seedlings has greater vulnerability to restrictive and adverse conditions (biotic and abiotic stresses) [60]. The use of potential microorganisms such as *Trichoderma* fungi can solve this problem and benefit the development of forest seedlings with mechanisms of action that improve plant growth, especially under adverse conditions. Under abiotic stress, *Trichoderma* benefits plant development by producing secondary metabolites, hydrolytic enzymes, phytohormones, and siderophores. Under biotic stresses, *Trichoderma* promotes biocontrol actions (parasitism, competition, and antagonism). In addition, members of this genus of fungi increase nutritional acquisition (production of secondary metabolites, phosphate solubilization, decomposition of organic matter) to promote greater availability of nutrients to plants.

**Figure 3:**
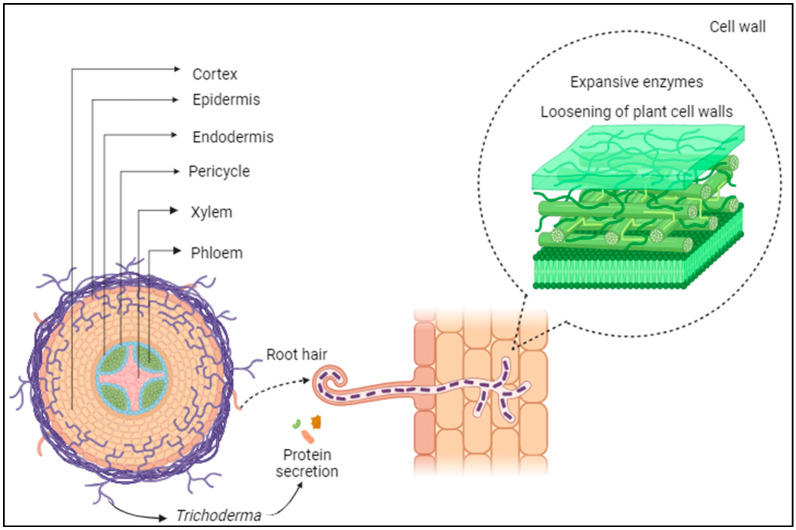
*Trichoderma* in fixation, penetration, and root colonization. Created in Biorender.

Figure 3 demonstrates that root colonization begins from the interaction of chemical signals between plant and fungus, composed of fixation, penetration, and root colonization, intermediated by the production of metabolites [46]. For the fungus to be able to colonize the roots, protein secretion must occur to loosen the plant cell wall and then facilitate root penetration and intercellular growth, limited to the epidermal layer and the external cortex [61]. The hydrophobic proteins, rich in cysteine, are involved in the initial bond with the root surface [62]; subsequently, the action of the expansive enzymes allows the loosening of plant cell walls through non-covalent interactions to maintain their integrity [63].

As a mechanism of interaction, the plants provide sucrose to the fungi *Trichoderma* to optimize their development and consequent root colonization. From this stage, after the mediation by hydrophobic proteins occurs, the installation and adhesion of *Trichoderma* on the plant root surface of host plants begins. These steps are important to the functioning of cell communication processes, fungal morphogenesis, and adherence of hyphae to hydrophobic surfaces [54,64].

Communication between forest seedlings and *Trichoderma* is complex and dynamic, based on the exchange and perception of chemical signals [65]. The extensive dialogue in the early stages of interaction releases the exudation of important compounds such as volatile organic compounds, secondary metabolites, and plant hormones [66]. Such substances greatly influence the seedling formation process, emphasizing the benefits from the seed germination process to the emergence of seedlings and seedling formation with high quality [7].

From there, the endophytic colonization begins, where the microorganism assists the development and growth of seedlings through the emission of chemical signals that allow the best plant development due to the symbiosis process. Consequently, shoot and root biomass are improved; seedlings also have greater resistance and responses to stress conditions [67,68].

Studies with the inoculation of *Trichoderma* in species of forest seedlings have shown positive effects on its development. In seedlings of *Acacia auriculiformis*, inoculation with strains of *Trichoderma* sp. increased plant total dry biomass compared to those not inoculated with the fungus [69]. In the production of argan seedlings (*Agrania spinosa*), after root colonization by *Trichoderma*, benefits were observed in the growth parameters through greater seed germination potential, root development, and plant height [70].

VOCs regulate the hormonal concentration of seedlings, which reflects in higher biomass production and yield through increased root volume [71], through the ability to induce better redistribution of auxins in the roots, and growth of seedlings [72]. The potential of SM infers significant gains in forest seedlings through increased seedling production [73]. In *Acacia mangium*, the inoculation of *T. viride* improved seedling dry matter, which can be explained by the higher absorption capacity of Fe by plants through the production of siderophores, VOCs, and secretion of hydrolytic enzymes [74,75].

Phytohormones (IAA, cytokinins, gibberellins) associated with SM synthesized by *Trichoderma* allow the increase of plant height and root development, as observed in *Camellia sinensis* [76]. In *Bougainvillea spectabilis*, soil treated with *T. longibrachiatum* stimulated root production in cuttings through the production of indole-3-butyric acid (IBA) and α-naphthalene acetic acid (NAA); such auxin regulators contribute to the development of morphological characteristics of seedlings, with emphasis on root development [77]. *T. virens* in oil palm seedlings (*Elaeis guineensis*) contributed to the increase in the production of growth factors and phytohormones by both isolates and consequent effective growth [78].

*Trichoderma* strains exude SM in olive seedlings (*Olea europaea*), which increases height, leaf area, stem diameter, and seedling clearance in nursery conditions [79,80]. One of the most promising functions performed by secondary metabolites refers to the multiplication of plant cells, in which the action performed by *Trichoderma* strains correlated with higher rates of germination speed and germination percentages and factors for obtaining seedlings with high morphological and physiological parameters [81].

The greater root development is one of the main effects provided by *Trichoderma* sp. because the growth of seedlings is optimized through the greater capacity of root exploration, which influences the wide capacity of absorption of macro and micronutrients of the soil [82]. The development of seedlings is optimized by the proper use of macro and micronutrients, with emphasis on nitrogen (N), phosphorus (P), potassium (K), and iron (Fe), required in the seedling formation stage, through changes in the anchoring of the root system or exudation of metabolites [83].

The root is the organ most susceptible to environmental variations, constituting a barrier to the survival of seedlings [84]. Some species of the genus *Trichoderma* are capable of excreting metabolites, auxin analogs, and other protein compounds around the root system, promoting the increase of primary and secondary roots, as well as stimulating the production of root hairs, which increase absorption of nutrients [85].

The fungi sequester the phosphate in the unavailable form in the soil through their mycelium and then release it to the seedlings in the readily available form [86]. This mechanism was observed in *Hevea brasiliensis*, where *Trichoderma* promoted the solubilization of insoluble phosphate into available phosphate due to the release of organic acid (citric acid), which promoted the development of rubber tree seedlings in the nursery [87,88].

Stimulating the development of the root system, height, and stem diameter contributes to the production of forest seedlings, with stem diameter in the seedling production phase being one of the desirable factors for reducing seedling time in nurseries [89]. In *Euterpe oleracea* seedlings inoculated with *Trichoderma*, there was interaction and consequent increases in stem diameter, which explains a greater capacity for survival of seedlings in the field [90].

Macro and micronutrients are involved in photosynthesis because such activity requires a series of chemical and physiological steps related to the adequate supply of nutrients [91]. As the interaction between *Trichoderma* and plant promotion occurs, seedling photosynthesis and total water content of *Quercus robur* L. avoid the reduction of energy during water transpiration [92]. In addition, reports are showing that photosynthetic capacity is high due to gene regulation developed by *Trichoderma* sp. to make the seedlings more resistant to adverse conditions and achieve higher-quality indexes [93].

During the initial stage of development of forest seedlings, they require adequate energy for greater efficiency in producing photoassimilates. During the growth of seedlings, the energy requirement is increased [94]. Plants synthesize carbohydrates through photosynthesis and obtain the energy content necessary for the breathing process, which is fundamental to the conduction of physiological activities for maintenance and growth of seedlings [95]. These strategies corroborate the success in the silvicultural sector destined to produce seedlings, providing favorable conditions to obtain more productive seedlings, a decisive stage for the good development of forest stands.

### Practical Examples of Trichoderma in the Formation of Forest Seedlings

The use of *Trichoderma* spp. in soil treatment is revealed as an alternative of great technological innovation, constituting a mechanism that promotes distinct gains in forest seedlings’ development, quality, and growth. The effectiveness in promoting plant growth comes from species capable of establishing lasting interactions with the plant, considering that the association is highly variable, whether in the function of the fungus species, development conditions, inoculum rate, or type of formulation [96]. Table 2 reports the symbiotic association between different species of *Trichoderma* and woody species capable of maximizing growth promotion.

The increase in dry mass is associated with the highest percentage of survival of seedlings at the time of transplanting, making seedlings tolerant to water restrictions, mediated by the ability to change the environment and promote prolonged mutualistic association [10,106]. The morphology of the seedlings directly affects their production potential since both the growth in the shoot and the root architecture depend upon the availability of nutrients in the soil [107]. The close relationship with plants ensures that *Trichoderma* fungi, when colonizing the root system, promote changes in plant metabolism, affecting plant growth and nutrition, the development of the root system, and the biocontrol of pathogens [9].

The action of microorganisms allows obtaining seedlings with a root system and well-developed shoot, which are determining factors for survival and desirable development in the seedling production process [108]. Furthermore, the increase up to 200% in the total plant biomass in inoculated plants indicates that the inoculation of *Trichoderma* is a promising method to produce seedlings at the commercial level [109]. The advantages of the use of potential microorganisms are due to the benefits at the physiological level, such as the increase in the photosynthetic potential, greater efficiency, and absorption of water and nutrients, which influence the arrangement of the morphological attributes of the seedlings [100,110].

Currently, the need to reduce the use of agrochemicals is increasing in sustainable agriculture. This plant–microorganism interaction is viable because, in addition to promoting the productivity of forest seedlings, growth promoters allow adding value to the product, making it more competitive and lower cost to the producer [55]. Remarkably, plant growth-promoting substances ensure improvements in the seed germination process and the development and quality of seedlings [8].

According to the morpho and physiological characteristics of the main forest species cultivated in Brazil, synergism via interactions with beneficial microorganisms becomes a useful tool for new microbial quality of the soil and forest production [111]. Since plant responses to fungi actions are broad, it is necessary to evaluate different conditions conducive to plant–microorganism interactions to improve silvicultural techniques for promoting seedling growth [112].

Promoting the growth of forest species through association with *Trichoderma* is shown to be a viable alternative for the sustainable production of seedlings in Brazil, which is capable of reducing the cost of production via less dependence on mineral fertilizers, has lower risks of environmental contamination, as well as speeds up the process of permanence in forest nurseries and possibly makes the seedlings more capable of being established in the field, according to improvements in the root system.

## 6. Potential Microorganisms: Co-Inoculation Capacity

**Figure 4:**
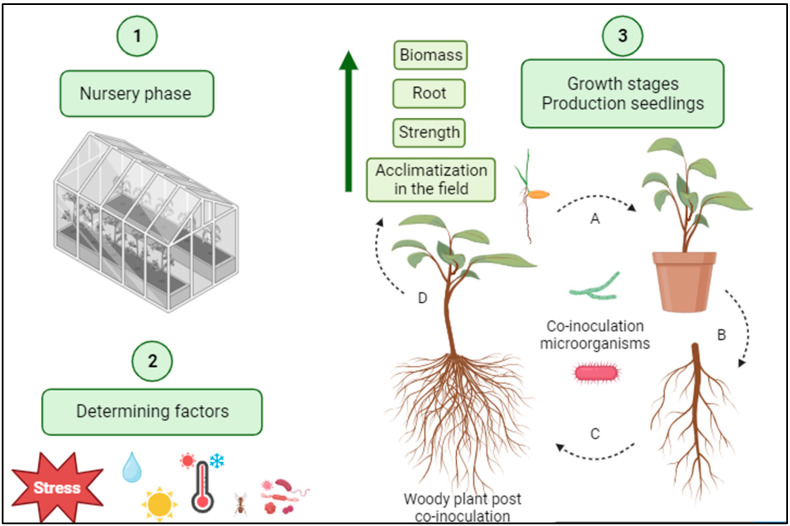
Capacity of microorganisms’ co-inoculation in the production process of forest seedlings. Stages: (1) Nursery phase mediated for (2) determining factors (biotic and abiotic stress), (3) growth stages in production seedlings co-inoculation ((A) better germination, (B) growth seedling, (C) root development, (D) fortified root, appearance of secondary roots and root hairs), as a result, obtaining seedlings with greater biomass, root system, strength and acclimatization in the field. Created in Biorender.

During the nursery phase (production of forest seedlings), several factors can influence positively or negatively the production capacity of plants, so it is essential to insert viable technologies to increase plant production (Figure 4). The system of “consortium” (combination of two or more microorganisms) segmented through the co-inoculation of microorganisms is a valuable alternative in the seedling development stage [113].

Despite the positive effects on the production and growth parameters of arboreal plants, biopromotion via double inoculation is variable, depending on the compatibility between such microorganisms and the amount of inoculum applied to avoid competition. Furthermore, the co-inoculation with fungi and bacteria can promote an antagonistic effect, which is undesirable in the silvicultural system production and sustainable development [114,115].

The interaction process between plant and microorganism is complex, which reinforces the need to evaluate the different effects of microbial consortium on plant development and forest seedling production. In contrast, each species presents specific characteristics and responses to interaction promoting additive/synergistic or antagonistic effects [77,116]. The beneficial effects of co-inoculation are observed in improvements of germination, vigor, root morphogenesis, photosynthetic capacity, and high biomass indexes, as well as enabling soil maintenance and ecological balance [117,118].

The success of communication and interaction between plants and microorganisms is observed in several species; in this sense, the evaluation of the potential of the process of co-inoculation between fungi and bacteria is a promising tool for the development of forest seedlings, as observed in the interaction between *Ambispora leptoticha*; *Azotobacter chroococcum* and *T. harzianum* under large-scale nursery conditions increasing teak biomass (*Tectona grandis*) [110]. In addition, in [119], some authors also obtained success for producing teak seedlings under co-inoculation with mycorrhizal fungi and rhizobacteria. In palm seedlings (*Elaeis guineensis*), the co-inoculation of *Bacillus cereus* and *T. asperellum* increased root growth and promoted greater plant development through phosphate solubilization [120].

Among the limiting factors to silvicultural development, the initial stages of seedling formation are highlighted to avoid a low increase in biomass and restrictions in the root system. Such factors have a direct association with the low nutrient content in the substrate used for the growth of seedlings; considering such a restriction, studies revealed that the co-inoculation of *Rhizoglomus fasciculatum* (arbuscular mycorrhizal fungus), *Mortierella* sp. (phosphate solubilizing fungus), and *Azospirillum brasilense* (plant growth promoter bacteria) promoted greater potential in the development and higher quality of seedlings [121].

Positive results were obtained through the consortium between microorganisms (*R. fasciculatus*, *A. chroococcum*, *B. coagulans*, and *T. harzianum*) in the production of seedlings of *Dalbergia sissoo*, where plants associated with microorganisms obtained high rates in all growth parameters, with good establishment and vigorous seedlings in the field [122]. Through the multifaceted action of microorganisms, co-inoculation is characterized as a promising method for the growth and development of seedlings [119].

The co-inoculation of mycorrhizal fungi (*R. irregulares*; *Funneliformis mosseae* and *Claroideoglomus etunicatum*) and *T. harzianum* increased shoot and root systems of apple trees, which led to a reduction in the need for nursery replanting, which are improvements resulting from the action of microorganisms in soil quality [123]. The mechanisms involved in promoting plant development are broad and have different facets under the conditions of cultivation, cultivated species, and action of the microorganism (synthesis of nutrients, phytohormones, mobilization of soil compounds), which influence and ensure that seedlings can develop in good conditions [124].

There are several microorganisms used as inoculum in tree seedlings, such as mycorrhizal arbuscular [125], growth-promoting bacteria [126], rhizobia [127], and *Trichoderma* [82]. These microorganisms improve root and shoot development and nutrient content in plants. The advantage of inoculating *Trichoderma* is that this genus is associated with plant growth, bioremediation, and the production of secondary metabolites.

## 7. Considerations and Future Perspectives

The inoculation of *Trichoderma* for seedling production in forest species promotes several benefits to plant development; in addition to the low production cost, it is a simple and effective practice, which stimulates root development, promotes greater nutritional absorption capacity, and increases plant biomass, which are determinant for obtaining more vigorous seedlings and greater economic yield.

Adopting beneficial microorganisms, such as fungi of the genus *Trichoderma*, is demonstrated not only as a viable strategy to produce seedlings of forest species but also as a sustainable alternative and recovery plan for degraded environments, from the considerable reduction in the use of agrochemicals and industrial fertilizers.

Another major advantage associated with the promotion of forest species through interaction with potential microorganisms is that most plant–microorganism interactions are observed in annual species, with few studies in the forest sector. There is a gap of understanding the different mechanisms of action of the symbiotic process, such as metabolic activity and mechanism of interaction with plants and other microorganisms, to increase the use of potential microorganisms in the silvicultural sector.

Although the great relevance and capacity to promote growth, there are still questions to be elucidated, such as the mechanisms involved in the solubilization of nutrients in potential native forest species, which would possibly enable its large-scale production.

Information correlated with the action of microorganisms in the silvicultural sector is still scarce, which requires greater attention.

## Figures and Tables

**Table 1:** Relationship between the production of chemical compounds by species of *Trichoderma* spp. and functions that promote growth.

Species	Chemical Production	Activity	References
*T. asperellum*; *T. harzianum*; *T. viride*; *T. koningii*	Siderophores	Fe chelating agents, in this process, Fe^3+^ siderophores are recognized and absorbed by plants, adopting a key role in the availability of the micronutrient	[46,47,48]
*T. harzianum*	Terpenes	Provides signals to plants that trigger changes in growth	[49,50,51]
*T. harzianum*	Metabolites of isocyanate	Positive impact on the symbiosis process	[50]
*T. harzianum*; *T. koningii*; *T. viride*	Pyrones	6-pentyl-2H-Piran-2-one (6-PP): chemical signaling via induction of auxin and ethylene formation, which modulates root architecture (formation of root hairs) Promotes seed germination and seedling development	[52,53,54]
*T. asperellum*; *T. harzianum*; *T. koningii*; *T. viride*	Synthesis of phytohormones	High rhizosphere competence. Performs the biosynthesis of indole-3-acetic acid (IAA), capable of modifying the root architecture and increasing root mass and rate of absorption of nutrients by the plant	[23,55]

**Table 2:** Relationship of growth promotion from the association between *Trichoderma* sp. and seedlings of forest species.

*Trichoderma* sp.	Forest Species	Effects	References
*T. harzianum*	*Abroma augusta*	Height and stem diameter	[97]
*T. harzianum*; *T. lignorum*; *T. koningii*	*Acacia mangium*	Height, stem diameter; biomass and root volume	[98]
*T. asperelloides*; *T. harzianum*	*Bauhinia forficata*	Height, stem diameter, and chlorophyll content	[99]
*T. asperelloides*	*Cabralea canjerana*	Height, biomass, and root system	[100]
*T. harzianum*	*Cedrela fissilis*	Height, biomass, and root system	[101]
*T. strigosellum*	*E. urophylla*	Height, number of leaves, and biomass	[102]
*T. asperellum*	*Enterolobium schomburgkii*	Height and stem diameter	[103]
*T. harzianum*	*Malus hupehensis*	Biomass and root system	[104]
*T. asperellum*	*Theobroma cacao*	Height and root system	[105]

## Data Availability

Not applicable.
